# Evaluation of Antioxidant Supplementation on Redox Unbalance in Hyperthyroid Cats Treated with Methimazole: A Blinded Randomized Controlled Trial

**DOI:** 10.3390/antiox9010015

**Published:** 2019-12-23

**Authors:** Alessia Candellone, Paola Badino, Paola Gianella, Flavia Girolami, Graziella Raviri, Vittorio Saettone, Giorgia Meineri

**Affiliations:** 1 Department of Veterinary Sciences, University of Turin. L.go Braccini, 2, 10095 Grugliasco (TO), Italy; alessia.candellone@unito.it (A.C.); paola.badino@unito.it (P.B.); paola.gianella@unito.it (P.G.); vittorio.saettone@unito.it (V.S.); giorgia.meineri@unito.it (G.M.); 2 Ambulatorio Veterinario Antica Reggia Della Dott.Ssa G. Raviri. Piazza V. Veneto, 3, 10078 Venaria (TO), Italy; ravvet@yahoo.it

**Keywords:** antioxidants, quercetin, curcumin, resveratrol, vitamin E, redox unbalance, feline hyperthyroidism, methimazole

## Abstract

Methimazole (MMI) is often the selected medical treatment for feline hyperthyroidism. However, the onset of MMI-related side effects (MMI-SE) is likely caused by oxidative stress. This study evaluated the dietary supplementation of selected antioxidants in hyperthyroid cats receiving MMI, to reduce MMI-SE. Thirty hyperthyroid client-owned cats were randomly allocated in group M (MMI + placebo) or group M+A (MMI + antioxidants). At different time-points from the enrolment (ET) to the end of the trial (FT), the following information was recorded: clinical findings, complete blood count, serum biochemical parameters, urinalysis, total plasma thyroxine concentrations, determinable reactive oxygen metabolites (dROMs), OXY-adsorbent test values, and oxidative stress index (OSi) values, and MMI-SE. dROMs and OSi values significantly increased from ET to FT in group M and were significantly higher in group M than in group M+A at FT. Likewise, OXY-absorbent test values were significantly higher in group M+A than in group M at FT. Moreover, the occurrence rate of MMI-SE in group M+A was lower than in group M. In conclusion, our results show that the dietary supplementation of antioxidants in hyperthyroid cats receiving MMI exerts a protective effect against oxidative stress, likely contributing to the reduction of MMI-SE.

## 1. Introduction

Feline hyperthyroidism is the most common endocrinopathy in middle-aged and geriatric cats sharing clinical, pathological, and therapeutic similarities with the human disease. As such, it offers a unique animal model for the study of human hyperthyroidism. [1]. Moreover, both species are at risk of developing redox unbalance during the disease course [2,3]. Previously, it has been demonstrated that hyperthyroid cats display impairment of the antioxidant status (AS) compared to both healthy cats and cats suffering from chronic non-thyroidal illnesses. Thus, they represent an ideal patient group for antioxidant supplementation [4]. Although non-curative, methimazole (MMI) is the medical treatment of choice for most hyperthyroid cats [5]. Unfortunately, the occurrence of MMI-related side effects (MMI-SE), such as hepatotoxicosis, gastrointestinal upset, vasculitis, facial pruritus, and blood dyscrasias, could hinder the attainment of euthyroidism. The onset of MMI-SE is correlated to an increase in oxidative stress (OS) markers in both humans and animal models [6]. Indeed, MMI metabolism generates intermediate reactive compounds, such as N-methyl thiourea and glyoxal, that can act as pro-oxidants both at cellular and tissue level [6,7]. Field studies in veterinary medicine are scattered, and clinical trials evaluating the effect of antioxidant supplementation during MMI treatment of hyperthyroid cats are lacking.

Among antioxidants, curcumin is one of the most used and effective hepato-protectant agents; resveratrol is able to ameliorate cardiovascular health [8,9]; quercetin inhibits thyroid type 1 deiodinase activity, and improves intestinal absorption and bioavailability of both curcumin and resveratrol [10,11]; and vitamin E plays a major role in destroying peroxyl radicals, thus protecting polyunsaturated fatty acids biological membranes from oxidative damage [12]. The concomitant supplementation of a mixture of such compounds in hyperthyroid cats receiving MMI could improve their clinical outcome and redox unbalance, reducing the risk of hepatic, cardiac, renal, and hematological alterations, elicited by both the disease itself and the antithyroid drug administration [13]. Thus, our study was designed first to evaluate the potential synergistic effect of curcumin, quercetin, resveratrol, and vitamin E as an additional treatment for hyperthyroid cats receiving MMI, and then to assess the capability of such molecules to reduce the occurrence of MMI-SE. 

## 2. Materials and Methods

**Figure 1:**
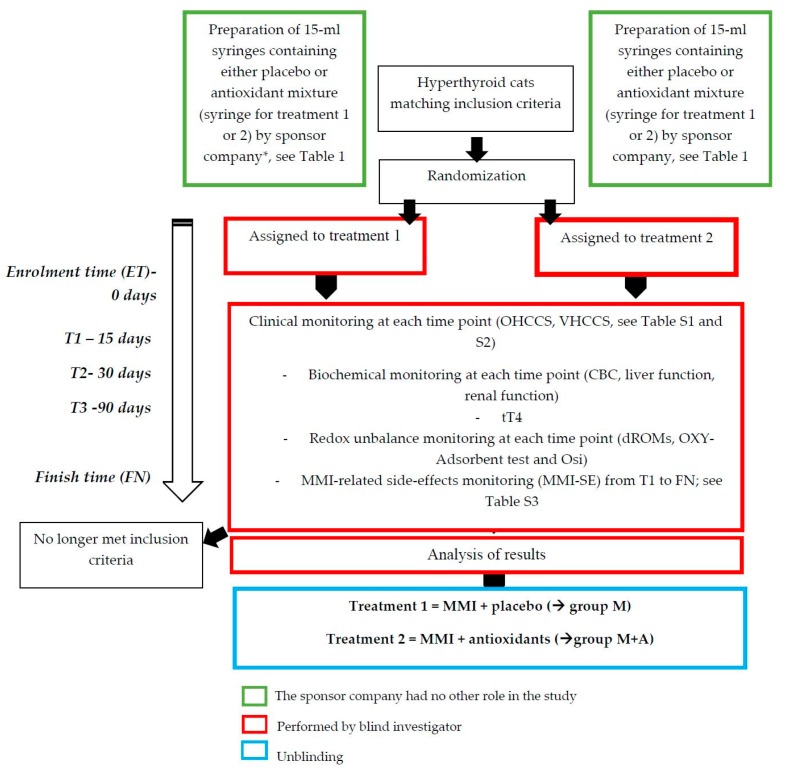
Schematic representation of the study design.

This study represents phase II of a previous research project [4]. All clinical maneuvers were performed in strict accordance with AAFP (American Association of Feline Practitioners) and ISFM (International Society of Feline Medicine) Feline-Friendly Handling Guidelines [14], were approved by the local bioethical committee of Turin University (EC 2017/42bis), and carried out in accredited ISFM Cat Friendly Clinics or in cat-friendly environments. All efforts were made to minimize animal discomfort. A written informed consent to participate was obtained from all cat owners. The study design is depicted in Figure 1.

### 2.1. Animals and Inclusion Criteria

From December 2017 to July 2018, newly diagnosed hyperthyroid cats presented to the Veterinary Teaching Hospital of the University of Turin and other referral clinics throughout Northern Italy were enrolled. Inclusion criteria were total plasma thyroxine (tT4) concentrations > 54 nmol/L or 4.3 µg/dL, as previously described, and owner agreement to administer the treatments and to attend all the scheduled appointments during the study [4]. Exclusion criteria were concurrent systemic diseases, such as congestive heart failure, renal failure (IRIS Stage 3 or 4) [15], systemic neoplasia, chronic liver disease, immune-mediated disease, or systemic infection [16], suspected nutritional deficiencies, antioxidant supplementation within 3-months, and administration of iodine-restricted food or commercial diet enriched with patented antioxidant formula [4]. 

### 2.2. Antioxidant Supplementation and Medical Treatment

Before starting the study, two sets of identical 15-mL graduated tube-syringes containing either a lipophilic emulsion (paste) of antioxidants (curcumin, quercetin, resveratrol, and vitamin E) or a lipophilic vehicle with a yellow inert pigment to mimic curcumin color (placebo) were commissioned from a sponsor company (DogPower, Novara, Italy). Antioxidants and their concentrations were selected according to the results obtained in previous in vitro experiments [17]. The composition of the antioxidant paste is reported in Table 1.

A double-blind study was performed dividing hyperthyroid cats into two treatment groups: one receiving MMI and placebo (group M), and one receiving MMI and antioxidants (group M+A). The allocation of animals into the two groups was performed by using a computer-generated random list (Microsoft Excel, Office 2011, Microsoft Corporation, Redmond, WA, USA). All cats were orally administered 1 mL/4 Kg/day of either placebo (group M) or the antioxidant paste (group M+A). From the enrolment (enrolment time, ET), each cat was monitored for 180 days (finish time, FT), with intermediate screenings after 15 (T1), 30 (T2), and 90 (T3) days, respectively, according to Daminet et al. [5].

All cats received a starting dose of 2.5 mg/animal MMI PO, administered twice daily. Drug dosing adjustments of 25%–50% increase or decrease were performed from T1 to FT, to obtain both plasma tT4 concentrations in the range of 1.0–2.8 μg/dL, and a proper control of clinical signs. Plasma tT4 concentrations were rechecked 2–3 weeks after every dosing adjustment.

### 2.3. Standardized Monitoring of Clinical Signs and Symptoms and MMI-SE

At ET, history and clinical findings were recorded through two ad hoc questionnaires, owner’s hyperthyroid cat clinical score (OHCCS) and veterinarian’s hyperthyroid cat clinical score (VHCCS), respectively (Tables S1 and S2). Briefly, the determination of body condition score (BCS) was assigned by considering the WSAVA 1–9 scoring system [18]. Feline body mass index (fBMI) was calculated by measuring the cat’s rib cage circumference (cm) and the lower back leg length (cm), from the knee to the ankle. Measures were then inserted into the “Cat’s Health—feline BMI App.” (mobile application for Windows Phone 8.1), and fBMI was automatically calculated. Modified semi-quantitative thyroid palpation was performed as previously reported [19]; non-invasive blood pressure measurement by using an oscillometric device (PetMap Plus+, Vetefarma, Madonna dell’Olmo, IT) was performed three times for each patient. To assess evidence of hypertensive retinopathy, ophthalmic examination by indirect ophthalmoscopy, was scheduled if systemic blood pressure (sBP) was >160 mmHg and diastolic blood pressure (dBP) was >100 mmHg. Blood samples were collected by jugular venipuncture for the evaluation of complete blood count (CBC), selected serum biochemical parameters (i.e., alanine aminotransferase, ALT; alkaline phosphatase, ALP; aspartate aminotransferase, AST; blood urea nitrogen, BUN; creatinine and phosphate) and plasma tT4 concentrations (IDEXX Catalyst One analysis, Milano, IT), as previously suggested [5]. Standard urinalysis (i.e., urine specific gravity, USG; dipstick analysis; sediment examination) was also performed.

From T1 to FT, MMI-SE were recorded by an ad hoc standardized questionnaire (side effects of methimazole treatment questionnaire, SEMT) (Table S3). In addition, CBC, the aforementioned biochemical parameters, and plasma tT4 concentrations were also monitored. The occurrence of liver dysfunction and hematological abnormalities was assessed by using VCOG-CTCAEv 1.0 guidelines [20]. In detail, hepatic toxicity was defined as moderate or severe if ALT measurement was ≥1.5–2.0 × ULN (upper limit of normal) or >2.0 × ULN, respectively, in association with moderate to severe clinical signs of hepatic dysfunction. Hematological abnormalities, such as neutropenia, thrombocytopenia, hemolytic anemia, and agranulocytosis, were considered only if severe clinical signs, related to blood dyscrasia, were detected.

Redox unbalance was determined from ET to FT by measuring determinable reactive oxygen metabolites (dROMs), OXY-adsorbent test values, and the oxidative stress index (OSi), as previously described [4]. All assays were purchased from Diacron International Srl (Grosseto, Italy). Briefly, d-ROMs were used as an indicator of oxidative stress due to free radicals, while the OXY-adsorbent test was used to quantify the plasma barrier against oxidation. The oxidative stress index (OSi) was calculated by the ratio of the dROMs test values to the OXY-adsorbent test values.

### 2.4. Statistical Analysis

To evaluate the differences between cats of group M and M+A for the nominal data (gender and breed) and ordinal data (BCS, OHCCS, VHCCS, and SEMT questionnaire scores), descriptive statistics analysis and the Pearson χ^2^ test were used. As regards SEMT scores, mild side effects were arbitrarily defined as scores ranging from 1 to 8 out of 25, moderate side effects from 9 to 16 out of 25, and severe side effects >17. At ET, differences between the two groups with respect to age, body weight, and USG were checked by *t*-test. Differences between the two groups at all time-points for CBC, the selected biochemical parameters, plasma tT4 concentrations, dROMs, OXY-adsorbent test values, OSi values, OHCCS, and VHCCS were tested by two-way ANOVA for repeated measures followed by Sidak’s post-hoc analysis considering all possible comparisons. All statistical analyses were performed using the software IBM SPSS V.20 (New York, NY, USA) and GraphPad Prism v7 (La Jolla, San Diego, CA, USA). Values of *p* ≤ 0.05 were considered statistically significant.

## 3. Results

Thirty-four cats were enrolled in the study. Four animals were excluded before the end of the trial due to either an intermittent administration of the treatment (*n* = 2; from group M+A) or the lack of compliance with the periodic controls (*n* = 2; from group M). Of the total 30 hyperthyroid cats that fulfilled the inclusion criteria, 14 were assigned to group M and 16 to group M+A. At ET, breed and gender distribution was homogeneous between groups and no statistically significant differences emerged with respect to age, body weight, BCS, OHCCS, VHCCS, CBC, selected biochemical parameters, USG, and redox unbalance (Table 2). In particular, average age of animals was equal to 12.8 and 12.9 years in group M and group M+A, respectively.

**Figure 2:**
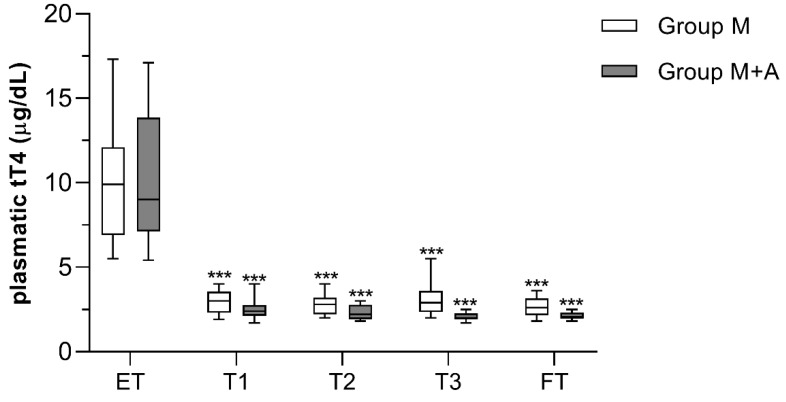
Total plasma T4 (tT4) concentrations in hyperthyroid cats receiving methimazole and placebo (group M, *n* = 14), or methimazole and antioxidants (group M+A, *n* = 16). Asterisks indicate statistically significant differences compared to ET (*** *p* < 0.001). Statistical analysis was performed by two-way ANOVA for repeated measures followed by Sidak’s post-hoc test. ET = enrollment time; T1 = 15 days; T2 = 30 days; T3 = 90 days; FT = finish time (180 days).

Similarly, statistically significant differences between the two groups in the mean plasma tT4 concentrations were not detected at any time-points. The values rapidly decreased to approximately 3 μg/dL (proper control of the disease) at T1 (*p* < 0.001 compared to ET) and remained almost unchanged until FT in all the cats (Figure 2). However, slight fluctuations of plasma tT4 concentrations were recorded in group M+A compared to group M throughout the study. Moreover, a lower MMI mean dose was administered to cats of group M+A compared to cats of group M (2.6 ± 0.6 mg/animal/twice daily vs. 3.3 ± 1.1 mg/animal/twice daily, respectively). Although not statistically significant, ALT values were more stable during time and across patients in group M+A than in group M, where some cats showed a moderate increase at FT (91.4 ± 25.8 IU/L vs. 121 ± 46.9 IU/L in group M, respectively. *p* = 0.052).

**Figure 3:**
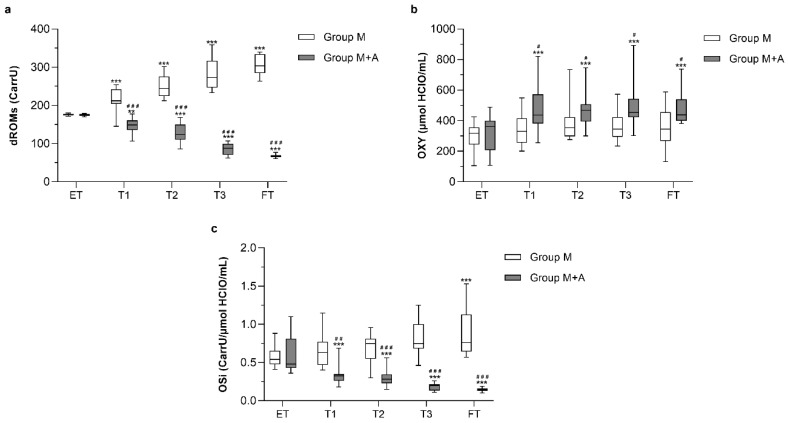
Determinable reactive oxygen metabolites (dROM) (**a**), OXI-adsorbent test (**b**), and OSi values (**c**) in hyperthyroid cats receiving methimazole and placebo (group M, *n* = 14), or methimazole and antioxidants (group M+A, *n* = 16). (** *p* < 0.01 and *** *p* < 0.001). Hashtags indicate statistically significant differences compared to group M (# *p* < 0.05, ## *p* < 0.01, and ### *p* < 0.001). Statistical analysis was performed by two-way ANOVA for repeated measures followed by Sidak’s posthoc test. ET = enrollment time; T1 = 15 days; T2 = 30 days; T3 = 90 days; FT = finish time (180 days)**.**

All data about redox unbalance (dROMs and OSi tests) and antioxidant capacity (OXY-adsorbent test) are summarized in Figure 3.

**Figure 4:**
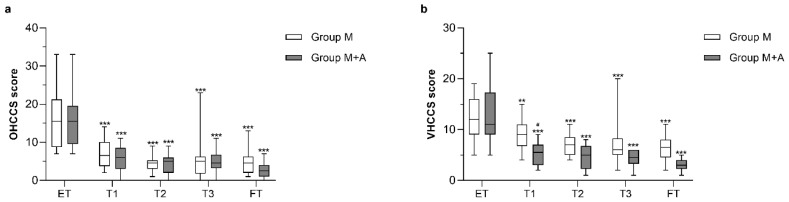
Owner’s hyperthyroid cat clinical score (OHCCS) (**a**) and veterinarian’s hyperthyroid cat clinical score (VHCCS) scores (**b**) in hyperthyroid cats receiving methimazole and placebo (group M, *n* = 14), or methimazole and antioxidants (group M+A, *n* = 16). Asterisks indicate statistically significant differences compared to ET (** *p* < 0.01 and *** *p* < 0.001). Hashtags indicate statistically significant differences compared to group M (# *p* < 0.05). Statistical analysis was performed by two-way ANOVA for repeated measures followed by Sidak’s post-hoc test. ET = enrollment time; T1 = 15 days; T2 = 30 days; T3 = 90 days; FT = finish time (180 days).

In detail, dROMs values progressively increased in group M up to 1.7-fold at FT, being statistically significant at all time-points compared to ET (*p* < 0.001). On the contrary, group M+A displayed a significant and time-dependent reduction of dROMs values up to 2.3-fold at FT (at least *p* < 0.01). Such a divergent temporal trend of free radicals production between groups was significantly related to treatment (*p* < 0.001 at all time-points). Consistently, OXY-adsorbent test values significantly increased only in group M+A (*p* < 0.001) and were significantly different from group M at all time-points (*p* < 0.05). As a consequence, OSi test results indicated an increase in oxidative stress in the M group compared to M+A at all time-points (at least *p* < 0.01). When considering OHCCS and VHCCS questionnaire scores, both groups showed a significant reduction of OHCCS and VHCCS from ET to FT (at least *p* < 0.01), with a significant difference between groups only at T1 (*p* < 0.05) (Figure 4). The occurrence rate of MMI-SE in group M+A was lower (12.5%) than in group M (35.7%). Indeed, five cats out of 14 exhibited at least one MMI-SE during the 180-days trial in the group M, compared to only two cats out of 16 in the group M+A. Only mild idiosyncrasies were recorded during both treatment protocols (100% of mild MMI-SE in group M+A vs. 80% of mild MMI-SE in group M).

## 4. Discussion

To the best of the authors’ knowledge, this study is the first clinical trial reporting the synergistic effect of a mixture of selected antioxidants as an additional treatment for hyperthyroid cats receiving MMI. Further, the potential ability to reduce the occurrence of MMI-SE is also considered.

Recently, it was demonstrated that hyperthyroid cats show an increased OS and a greater impairment of antioxidant capacity in comparison with healthy cats and cats diagnosed with chronical non-thyroidal illnesses. Moreover, it was proposed that hyperthyroid cats may serve as a spontaneous animal model for OS-related studies linked to thyrotoxicosis, representing an ideal cohort for nutritional and antioxidant interventions [4]. Although the link between MMI-SE and redox unbalance has been acknowledged in both humans and laboratory animal models [6,7], comparative studies in domestic species are scarce. Signalment, thyroid status, and hemato-biochemical values of cats of the present trial were consistent with previous data [13,16]. With respect to the OS induced by hyperthyroidism, our findings were consistent with previous studies both in veterinary and human medicine [2,4].

On the contrary, the only trial investigating the redox status of hyperthyroid cats before and after radioiodine treatment did not show any AS impairment irrespective of therapy [16]. However, it must be noted that the investigated parameters (i.e., reduced GSH, ascorbate, vitamin A, and vitamin E) mirrored only a fraction of the antioxidant plasmatic barrier, which is actually assessed by the OXY-adsorbent test. Moreover, the only investigated OS marker was the urinary isoprostanes level, which resulted significantly increased in hyperthyroid cats and was normalized upon treatment. No association was found between OS and prior idiosyncratic MMI toxicosis [16]. In our study, both groups of hyperthyroid cats were experiencing redox unbalance at recruitment (mean OSi value of group M and group M+A at ET: 0.66 ± 0.3 CarrU/µmol HClO/mL); such condition worsened in patients receiving MMI alone (mean OSi value of group M at FT: 1.01 ± 0.5). These results suggest that the redox unbalance could be induced not only by the thyroid dysfunction but also by the administration of MMI itself. Indeed, in group M+A, the attainment of the euthyroid state was paralleled by both a progressive decrease in dROMs values at all time-points and a slight increase in OXY-Adsorbent test values at T1, which remained constant until the end of the study (on average around 470 µmol HClO/mL). T1 can be considered a sort of “watershed” moment in the fluctuation of AS markers in both group M and group M+A (Figure 4). Noteworthy, the onset of MMI-SE is usually described from 2 to 6 weeks after the beginning of the treatment [5,21,22,23,24,25]. Interestingly, the increase in dROMs and OSi levels in group M occurred at the same time point, when it is also likely that the production of MMI reactive metabolites challenges the endogenous antioxidant defenses. Thus, the antioxidant supplementation might represent a supportive intervention to reduce the progressive deterioration of the endogenous antioxidant barrier.

The occurrence of MMI-SE in the present study was relatively higher in both groups (35.7% in group M and 12.5% in group M+A) compared to a previous trial [21]. However, it should be noted that the MMI-SE were mostly mild or moderate, and mainly related to occasional gastrointestinal signs (such as vomiting or diarrhea) and lethargy, rather than life-threatening complications. The relative overestimation of drug-adverse reactions can be explained by the accurate and standardized monitoring that could have highlighted events otherwise unnoticed. Although studies reporting a standardized evaluation of clinical signs and findings are lacking, the OHCCS and VHCCS results underlined that MMI alone or in association with antioxidants is efficient in reducing clinical symptoms of thyrotoxicosis in both groups, with a slight better clinical outcome in group M+A.

The MMI dose administered to all cats is in the range reported by other authors [24,25]. However, group M+A received a lower MMI mean dose compared to group M. A possible explanation of such phenomenon might be related to quercetin, one of the antioxidants contained in the mixture, reported to inhibit thyroid peroxidase (TPO) iodination in vitro and to modulate several enzymes involved in the synthesis and catabolism of thyroid hormones [26]. 

Although all the commercial diets administered to the cats were checked for both the nutritional adequacy and the lack of antioxidant patented formula in their composition, the study was performed with an unstandardized diet. Moreover, precise levels of vitamin E (usually used as a preservative) were not measured, assuming that they were within FEDIAF suggested requirements [27]; thus, different intake between the two groups could not be excluded. Nevertheless, the level of supplementation adopted in the present study was around 4-fold the minimum requirements; thus, vitamin E levels fluctuation due to different dietary uptakes is reasonably irrelevant.

## 5. Conclusions

In conclusion, we report here that a mixture of antioxidants, namely curcumin, quercetin, resveratrol, and vitamin E, can be efficiently administered to hyperthyroid cats to exert a synergistic effect with MMI. The amelioration of the clinical pathological outcome likely results from both the rebalancing of the redox status impaired by the disease, and the reduction of MMI-SE through the restoration of the antioxidant plasmatic barrier. However, additional studies with a higher number of animals are needed to further explore the role of antioxidant dietary supplementation in the treatment of feline hyperthyroidism.

## Figures and Tables

**Table 1:** Composition of the 15-mL syringe containing the antioxidant mixture formulated as a paste.

Antioxidant Compound *	Dose in 1mL of Paste
Curcumin	100 mg
Quercetin	40 mg
Resveratrol	1.5 mg
Vitamin E	15 mg

* Inactives: sunflower oil, malt, soybean oil, glyceryl stearate, lecithin e322.

**Table 2:** Clinical pathological variables in hyperthyroid cats of group M (MMI + placebo) and group M+A (MMI + antioxidants) at ET. Values are expressed as mean values ± standard deviation (SD).

Clinical pathological Features	Parameters	Group M (*n* = 14)	Group M+A (*n* = 16)	Reference Interval
**Signalment**	Age (years)	12.8 ± 2.4	12.9 ± 2	/
	Breed (*n*)	DSH 9; DLH 3; PERS 1; CHART 1	DSH 11; DLH 1; PERS 1	/
	Gender (*n*)	MC 6; FN 8	MC 6; FN 10	/
	Body Weight (kg)	3.9 ± 0.7	4.0 ± 0.8	/
	BCS	4 ± 0.5	4 ± 0.5	0-9
**Hemato-biochemical values**	RBC (×10^6^ /uL)	6.6 ± 1.3	6.5 ± 1.1	4.6–10
	Htc (%)	34.2 ± 6.7	31.6 ± 6.2	28–49
	WBC (×10^3^ /uL)	11.5 ± 5.3	11 ± 4.5	5.5–19.5
	Alb (g/dL)	3 ± 0.8	3.3 ± 0.8	2.2–4.4
	BUN (mg/dL)	22.7 ± 5.8	23 ± 5.8	10–30
	CREA (mg/dL)	1.3 ± 0.3	1.3 ± 0.3	0.3–1.6
	ALT (UI/L)	93.7 ± 23	92.1 ± 22.1	20–100
	AST (UI/L)	89 ± 27	91 ± 18	10–100
	ALP (UI/L)	42 ± 8.4	39 ± 9.6	10–50
	Phos (mg/dL)	4.8 ± 1.5	5 ± 1.9	2.4–8.2
	Urinary SG	1039 ± 14	1041 ± 12.1	>1035
**Thyroid function**	tT4 (µg/dL)	10 ± 3.4	10.3 ± 3.7	0.8–4.3
**Clinical scores**	OHCCS	16.1 ± 7.4	16.4 ± 7.4	0–35
	VHCCS	12.1 ± 4	12.6 ± 5.7	0–30
**Redox unbalance**	dROMs (CarrU)	175.8 ± 2.2	175.3 ± 2.6	<104 *
	OXY-Adsorbent (µmol HClO/mL)	301.4 ± 84	321 ± 115	>390 *
	OSi (CarrU/µmol HClO/mL)	0.66 ± 0.3	0.66 ± 0.3	<0.27 *

Alb: albumin; ALP: alkaline phosphatase; ALT: alanine aminotransferase; AST: aspartate aminotransferase; BCS: body condition score; BUN: blood urea nitrogen; CHART: Chartreux; CREA: creatinine; DLH: domestic longhair cat; DSH: domestic shorthair cat; ET: enrollment time; FN: neutered female; GLU: glucose; Htc: hematocrit; MC: male castrated; OHCCS: owner’s hyperthyroid cat clinical score; PERS: Persian; Phos: phosphate; RBC: red blood cells; SD: standard deviation; SG: specific gravity; tT4: plasma total tetraiodothyroxine; VHCCS: veterinarian’s hyperthyroid cat clinical score; WBC: white blood cells. *see [4].

## Supplementary Materials

The following are available online at https://www.mdpi.com/2076-3921/9/1/15/s1, Table S1: Standardized questionnaire formulated *ad hoc* for the evaluation of clinical signs and symptoms of hyperthyroid cats, as reported by the owner (Owner Hyperthyroid Cat Clinical Score, OHCCS), Table S2: Standardized questionnaire formulated ad hoc for the evaluation of clinical signs and symptoms of hyperthyroid cats, as reported by the veterinarian (Veterinarian Hyperthyroid Cat Clinical Score, VHCCS), Table S3: Standardized questionnaire formulated ad hoc for the evaluation of MMI-related side effects (Side Effects of Methimazole Treatment, SEMT).
