# Supplementary material for: Evaluation of Antioxidant Supplementation on Redox Unbalance in Hyperthyroid Cats Treated with Methimazole: A Blinded Randomized Controlled Trial
Source: Antioxidants (Basel). 2019 Dec 23;9(1):15. doi: 10.3390/antiox9010015 (PMC7022672; doi:10.3390/antiox9010015)
Supplement: Supplementary file 1 [file antioxidants-09-00015-s001.pdf]

Table S1. Standardized questionnaire formulated *ad hoc* for the evaluation of clinical signs and symptoms of hyperthyroid cats, as reported by the owner (Owner Hyperthyroid Cat Clinical Score, OHCCS).

	OHCCS (Owner Hyperthyroid Cat Clinical Score)					
	**0**	**1**	**2**	**3**	**4**	**5**
Polyphagia	Not reported; the cat shows unmodified feeding behavior	Request of extra-food before or after feeding time	Occasional request of extra-food during the day and before or after feeding time	Frequent request of extra-food during the day and before or after feeding time	Constant request of extra-food during the day and before or after feeding time	Constant request of extra-food all day and night long
Weight loss	Not reported	Weight loss clinically undetectable but detectable on the board	Mild weight loss clinically detectable (≈ 2–3 % of BW)	Moderate weight loss clinically detectable (≈ 4–5 % of BW)	Severe weight loss clinically detectable (> 5 % of BW)	Extreme weight loss clinically detectable with muscle wasting (> 10 % of BW)
PU/PD	Not reported	Greater in or nocturia up to 2xnormal, weekly reported	Greater in or nocturia up to 2Xnormal, daily reported	Urine production >2Xnormal but < hourly. Frequent search of water (water consumption still in the normal range)	>1 mL $Kg^{-1}h^{-1}$ urine production > 2 mL Kg^{-1} h^{-1}. Constant search of water (water consumption > 45 mL/kg/day	<3 mL $Kg^{-1}h^{-1}$ urine production. Constant search of water (water consumption >60 mL/kg/day
Vomiting/Diarrhea	Not reported	Vomiting and/or diarrhea reported one or two times monthly, self-limiting. Consultation and supportive therapy not needed	Vomiting and/or diarrhea reported one or two times weekly. Consultation needed without home therapy	Vomiting and/or diarrhea reported > 2 times weekly or on a daily base. Consultation an home therapy needed	Diarrhea or vomiting reported >3 times on a daily base; hospitalization and supportive care needed	Diarrhea or vomiting reported several times on a daily base; hospitalization and intensive care needed. Life-threatening condition
Behavioral changes (aggressiveness, irritability, panic attacks…)	Not reported; the cat shows unmodified social behavior	Occasional signs of irritability and discomfort during social interactions, without showing an aggressive behavior	Frequent signs of irritability and discomfort during social interactions, with few episodes of aggressive behavior	Constant signs of irritability and discomfort during social interactions, with frequent episodes of aggressive behavior	As score 3 + changing in social hierarchy with other pets and or altered interactions with the owner. Few episodes of vocalization during the night	Several episodes of aggressive behavior on a daily base; total subversion in social hierarchy, absence of interactions with owners, vocalizations all night long
Anorexia	Not reported; the cat shows unmodified feeding behavior	Oral intake always present but with signs of disorexia and capricious appetite; dietary change not needed.	Oral intake altered (1 day duration); dietary change required to maintain appetite	Oral intake altered (1–3 days duration) but no hospitalization needed. Oral nutritional supplements/appetite stimulants may be indicated	Oral intake severely altered (>3 days duration); hospitalization and feeding tube needed.	Oral intake severely altered (>5 days duration); hospitalization and intensive care required. TPN indicated. Life-threatening condition
Lethargy	Not reported; the cat shows unmodified ADL	Occasional or intermittent lethargy over baseline weekly reported; unmodified ADL	Mild lethargy over baseline; diminished spontaneous activity not causing difficulties in performing ADL	Moderate lethargy causing some difficulties with performing ADL; ambulatory only to point of eating, sleeping and litterbox area	Severely restricted in ADL; unable to confine urination and defecation to litterbox area. Food consumed only if offered in place	Diabled, must be force fed and helped to perform ADL

ADL: activity of daily living; BW: body weight

OHCCS (Owner Hyperthyroid Cat Clinical Score) / Clinical chart						
**CASE NUMBER/ PATIENT SIGNALMENT**	**# ___ Owner ______ Cat name _____ Breed______ Age ____ BW___ Life-style____ Feeding _______**					
**Date:**	**ET**	**T1**	**T2**	**T3**	**FT**	**Comments**
Polyphagia						
Weight loss						
PU/PD						
Vomiting/Diarrhea						
Behavioral changes (aggressiveness, irritability, panic attacks…)						
Anorexia						
Lethargy						
**OHCCS-TOTAL SCORE:**						

ADL: activity of daily living; BW: body weight

Table S2. Standardized questionnaire formulated *ad hoc* for the evaluation of clinical signs and symptoms of hyperthyroid cats, as reported by the veterinarian (Veterinarian Hyperthyroid Cat Clinical Score, VHCCS).

**VHCCS (Veterinarian Hyperthyroid Cat Clinical Score)**						
	**0**	**1**	**2**	**3**	**4**	**5**
Modified BCS	BCS = 7or >, fMBI in the Over weight area	BCS = 6, fBMI in the Normal area	BCS = 5, fBMI in the Normal area	BCS = 4, fBMI in the Normal or Under weight area	BCS = 2 or 3, fBMI in the Underweight area	BCS = 1, severe muscle wasting, fBMI in the Underweight area
Coat and skin status	Coat extremely well kempt, clean and shiny	Coat well kempt, clean and shiny	Coat kempt enough, clean but opaque	Coat poorly kempt, opaque but still clean	Unkempt coat	Skin lesions, alopecic areas
Attitude toward clinical maneuver	Collaborative and friendly patient which can be handled with minimum restraint	Collaborative patient which need some restraint	Tolerant patient which need restraint	Poorly tolerant patient which shows early signs of annoyance. Firm restraint needed	Intolerant patient which shows immediate signs of annoyance. Firm restraint needed.	Intolerant and aggressive patient which need restraint devices to be examined
Ocular fundic examination focused on hypertensive lesions (Performed only when mean systolic blood pressure was > 160 mmHg and diastolic blood pressure was > 100 mmHg)	Not reported	Vomiting and/or diarrhea reported one or two times monthly, self-limiting. Consultation and supportive therapy not needed	Vomiting and/or diarrhea reported one or two times weekly. Consultation needed without home therapy	Vomiting and/or diarrhea reported > 2 times weekly or on a daily base. Consultation an home therapy needed	Diarrhea or vomiting reported >3 times on a daily base; hospitalization and supportive care needed	Diarrhea or vomiting reported several times on a daily base; hospitalization and intensive care needed. Life-threatening condition
Cardiac auscultation	Normal heart rate and heart sounds	Mild to severe tachycardia (<200 bpm) with normal heart sounds	Severe tachycardia and "pounding" heart sounds	Heart murmur	Gallop rhytm	Signs of cardiac heart failure
Modified semi-quantitative thyroid palpation	Equivalent to score 0 according to Norsworthy et al., 2002	Equivalent to score 1 according to Norsworthy et al., 2002	Equivalent to score 2 according to Norsworthy et al., 2002	Equivalent to score 3 according to Norsworthy et al., 2002	Equivalent to score 4 according to Norsworthy et al., 2002	Equivalent to score 5 and 6 according to Norsworthy et al., 2002

BCS: Body condition score; fBMI = feline Body Mass Index.

**VHCCS (Veterinarian Hyperthyroid Cat Clinical Score) / Clinical chart**						
**CASE NUMBER/ PATIENT SIGNALMENT**	# ___ **Owner** ______ **Cat name** _____ **Breed**______ **Age** ____ **BW**___ **Life-style**____ **Feeding** _______					
**Date:**	**ET**	**T1**	**T2**	**T3**	**FT**	**Comments**
Modified BCS						
Coat and skin status						
Attitude toward clinical mannouvre						
Ophtalmic examination						
Cardiac auscultation						
Modified semi-quantitative thyroid palpation						
**VHCCS-TOTAL SCORE:**						

Table S3. Standardized questionnaire formulated ad hoc for the evaluation of MMI-related side effects (Side Effects of Methimazole Treatment, SEMT).

SEMT (Side-effects Methimazole Treatment)						
	**0**	**1**	**2**	**3**	**4**	**5**
Anorexia	Not reported; the cat shows unmodified feeding behavior	Oral intake always present but with signs of disorexia and capricious appetite; dietary change not needed.	Oral intake altered (1 day duration); dietary change required to maintain appetite	Oral intake altered (1–3 days duration) but no hospitalization needed. Oral nutritional supplements/appetite stimulants may be indicated	Oral intake severely altered (>3 days duration); hospitalization and feeding tube needed.	Oral intake severely altered (> 5 days duration); hospitalization and intensive care required. TPN indicated. Life-threatening condition
Lethargy	Not reported; the cat shows unmodified ADL	Occasional or intermittent lethargy over baseline weekly reported; unmodified ADL	Mild lethargy over baseline; diminished spontaneous activity not causing difficulties in performing ADL	Moderate lethargy causing some difficulties with performing ADL; ambulatory only to point of eating, sleeping and litterbox area	Severely restricted in ADL; unable to confine urination and defecation to litterbox area. Food consumed only if offered in place	Disabled, must be force fed and helped to perform ADL
Vomiting/Diarrhea	Not reported	Vomiting and/or diarrhea reported one or two times monthly, self-limiting. Consultation and supportive therapy not needed	Vomiting and/or diarrhea reported one or two times weekly. Consultation needed without home therapy	Vomiting and/or diarrhea reported > 2 times weekly or on a daily base. Consultation and home therapy needed	Diarrhea or vomiting reported >3 times on a daily base; hospitalization and supportive care needed	Diarrhea or vomiting reported several times on a daily base; hospitalization and intensive care needed. Life-threatening condition
Facial pruritus/excoriation	Not reported	Occasional (reported on weekly base), not interfering with feeding and sleeping activity	Intermittent (>3 episodes reported per week), not interfering with feeding and sleeping activity	Mild but prolonged (reported on daily base), partially interfering with sleeping but not with feeding activity	Moderate but prolonged, partially interfering with feeding and sleeping activity	Severe and prolonged, continues when the cat is eating or sleeping. Signs of self-mutilations
Liver dysfunction	Non reported	Light signs attributable to hepatic dysfunction	Mild signs attributable to hepatic dysfunction + jaundice	Moderate signs of acute liver failure. Hospitalization and supportive care needed.	Severe signs of acute liver failure. Hospitalization and intensive care needed. Life-threatening condition	Hepatic encephalopathy or coma

TPN =Total Parenteral Nutrition; ADL =Activity of daily living

SEMT (Side-effects Methimazole Treatment) / Chart						
**CASE NUMBER/ PATIENT SIGNALMENT**	# ___ **Owner** ______ **Cat name** _____ **Breed**______ **Age** ____ **BW**___ **Life-style**____ **Feeding** _______					
**Date:**	**ET**	**T1**	**T2**	**T3**	**FT**	**Comments**
Anorexia						
Lethargy						
Vomiting/Diarrhea						
Facial pruritus/excoriation						
Liver dysfunction						
**SEMT-TOTAL SCORE:**						
